# Deploying artificial intelligence for occupational and environmental health in waste management: a systematic review

**DOI:** 10.3389/ijph.2026.1610020

**Published:** 2026-09-21

**Authors:** Wei Li, Zhengyan Ge, Hui Wang, Mingzi Yuan, Zhican Yin

**Affiliations:** 1 College of Public Administration, ASEAN Research Center, Huazhong University of Science and Technology, Wuhan, China; 2 School of Economics, Huazhong University of Science and Technology, Wuhan, China

**Keywords:** environmental health, institutional innovation, occupational safety, responsible artificial intelligence, waste management

## Abstract

**Objectives:**

This systematic review examines how artificial intelligence (AI) mitigates occupational and environmental health risks in waste management, a sector where workers and communities face infectious, toxic, and physical hazards.

**Methods:**

Following PRISMA guidelines, we systematically searched the SCOPUS database and reviewed 81 peer-reviewed articles. The analysis focused on the types of AI technologies applied, their deployment across waste management stages, and the specific health risks they address.

**Results:**

The analysis identifies five types of health risks targeted by AI applications. AI is deployed across five waste management stages and four technical paradigms (supervised, unsupervised, reinforcement, and deep learning). AI alleviates hazards via three primary pathways: automated risk-source isolation, robotic substitution for high-risk tasks, and macro-level exposure monitoring.

**Conclusion:**

Current public health research has largely neglected health risk reduction gains from waste management technological innovation. Institutional innovations that can facilitate responsible AI deployment are discussed. This interdisciplinary perspective underscores the necessity of cross-sector collaboration to tackle public health issues.

## Introduction

### Background

With the continuous advancement of global industrialization and urbanization, the volume of solid waste is growing rapidly while recycling rates remain low in developing countries [1], becoming a significant issue affecting ecological security, public and occupational health, and sustainable development. According to the Lancet Commission [2], waste pollution management is explicitly or indirectly related to several United Nations’ Sustainable Development Goals: SDG1 (no poverty), SDG3 (good health and wellbeing), SDG6 (clean water and sanitation), SDG10 (reduced inequalities), SDG11 (sustainable cities and communities), SDG13 (climate action), SDG14 (life below water) and SDG15 (life on land). There is an urgent need to break through the limitations of traditional management models through technological innovation.

According to the International Labor Organization [3], occupational health risk refers to the combined likelihood of an occurrence of a hazardous event and the severity of injury or damage to the health of employees or workers caused by this event. Environmental health risks can be broadly defined as the chances of harmful effects to human health and/or to ecological systems resulting from exposure to an environmental stressor, including any physical, chemical, social, or biological entity that can induce a change in health, wellbeing, and quality of life [4]. Certain occupational health risks can also be environmental health risks.

Traditional waste management system primarily adopts a “collection-transportation-landfill/incineration” model, with a high proportion of manual sorting [5]. The traditional technologies have high misclassification rates [6], and struggle to handle complex waste forms in disaster scenarios. These problems have triggered two types of health risks. The first type is direct exposure risks to workers and nearby residents during stages of source separation, collection and waste treatment [7, 8]. These risks are particularly prominent in informal waste recycling systems lacking effective health protection. The second type are risks primarily affecting broader populations through cross-media migration of pollutants. According to the World Health Organization [9], poor waste treatment is associated with health outcome such as cancer and respiratory illness, impaired child neural development and chronic stress.

Since the 1990s, with a paradigm shift from “end-of-pipe disposal” to “whole-life-cycle collaborative governance”, source reduction, separate collection, and material recycling becoming core issues in waste management [10]. AI technology has emerged as a critical solution to address these challenges. Existing reviews of AI technology in waste management have mainly focused on cost-efficiency and technical optimization, with limited coverage of associated public health implications [11, 12]. Yevle and Mann [13] noted through a bibliometric analysis of 75 studies that AI research in waste management remains fragmented, and the integration of informal sectors is overlooked.

Furthermore, mainstream public health literature largely ignores the public health benefits generated by technological innovations within waste management systems. This article advances the literature by providing a systematic review of how AI-driven innovation in “whole-life-cycle” waste management mitigates direct and indirect health hazards for workers, residents, and communities. It foregrounds the ethical considerations and socio-technical dimensions of AI deployment in this domain—issues that remain under-explored in prior review studies. Its interdisciplinary lens helps policymakers expand their public health intervention strategies and attach greater importance to deploying AI in waste management as a public health instrument.

### AI technologies in waste management

In the context of accelerated global urbanization and industrialization, solid waste management has evolved into a dual challenge for environmental governance and public health [14, 15]. Especially in developing areas, uncontrolled open stacking and simple landfills are still common due to infrastructure and institutional bottlenecks, posing a long-term threat to the health of surrounding communities [16–18]. In this system, frontline sanitation workers and informal scavengers are in the most critical situation, facing serious toxicological risks and respiratory damage when manually handling hazardous household waste and electronic waste [19, 20].

To address these risks, the introduction of AI technologies is seen as a key initiative to drive the industry’s transformation to intelligence and safety [21]. Existing research has proven that the separation of human labor from hazardous environments can be achieved through deep learning visual recognition technologies. The combination of convolutional neural network and automatic robot sorting system realizes high-precision automation substitution in many high-risk scenarios [22, 23]. In addition, advanced AI algorithms can accurately predict the amount of municipal waste generated, minimizing secondary pollution stemming from waste accumulation during waste collection logistics and resource recovery processes [24–26].

However, there is still a gap between technologies and applications. Although AI has significantly improved technical and cost efficiency, scholars pointed out that existing models lack sufficient robustness when facing highly heterogeneous and complex waste flows in real-life scenarios [21]. While theoretical framework unifying hazardous waste management and health risks has used simulations to assess feasibility, real-world validation is lacking [27]. Current optimization algorithms tend to focus on technical indicators (such as accuracy, cost, path) and lack a “health-centered design concept” [28].

## Methods

This study adheres to the PRISMA (preferred reporting items for systematic reviews and meta-analyses) guideline to ensure a rigorous and transparent review [29]. The step-by-step process has been presented in Figure 1. In all steps of the review process, five authors are involved in searching, screening and coding the articles. The first author identified abnormality in the database and removed irrelevant articles searched by the other four authors. As the research topic is relatively under-researched, the review includes both empirical studies, review studies and conceptual research of AI technology applications in various stages of waste management. The SCOPUS database was used for retrieving research articles, which includes peer-reviewed journals in multiple disciplines and provides advanced search tools.

**FIGURE 1:**
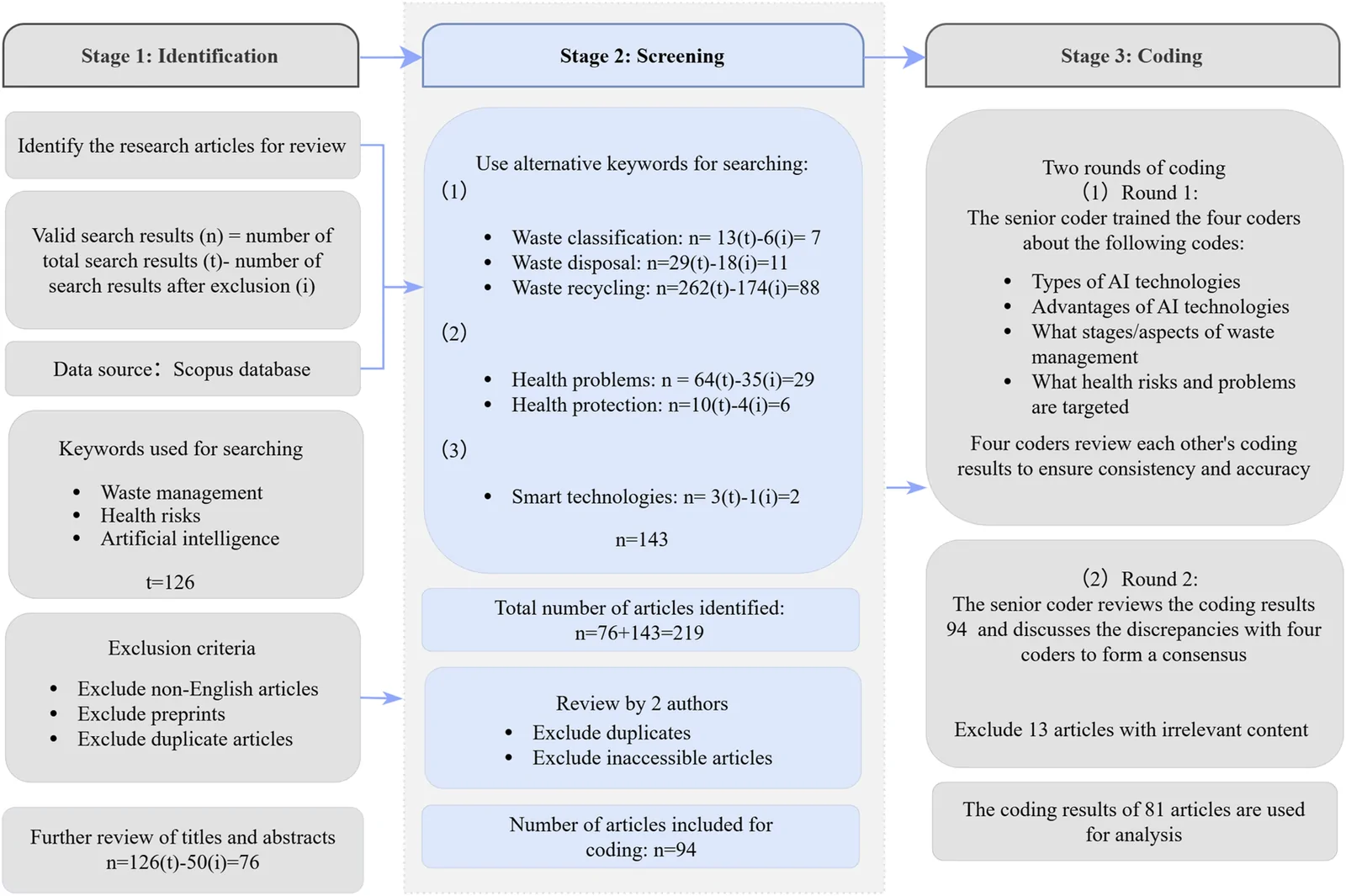
The process of collecting review articles (China, 2026).

In Stage I (April 2025), the authors used keywords “waste management” and “health risk” to search research articles in the SCOPUS, and used the keyword “artificial intelligence” to refine the search results. A total of 126 articles were identified. After excluding non-English articles, preprint studies and duplicated articles, the authors reviewed the titles and abstracts, and retained 76 relevant articles.

In Stage II (May 2025), we used additional keywords following the same procedure in stage I, to ensure the comprehensiveness of the review.

Firstly, the authors adopted the same search procedure using the keyword “waste classification”, yielding 13 articles, of which 7 were relevant. Then the keyword “waste disposal” was used for searching, resulting in 29 articles, of which 11 were relevant. We also used “waste recycling” and identified 252 articles and 10 preprints. 88 relevant studies were retained in this step. Secondly, the authors used the keyword “health issue” for searching, which yielded 59 articles and 5 preprints. After screening, 29 articles were identified. Using “health protection” for searching, we added 6 articles for review. Thirdly, the authors used “smart intelligence” and identified additional 2 relevant articles. In total, 143 additional relevant articles were identified after this stage, bringing the number of articles selected for review to 219. Two authors further cleaned the pool of selected articles. After removing duplicated articles and the articles that are not downloadable, 94 articles were included in the review.

In Stage III (October 2025), the authors conducted two rounds of coding. In the first round, one senior author trained four authors who then shared the task of coding 94 articles. The coding items that are analyzed include “types of AI technologies, advantages of AI technologies, AI technologies applying to what stages/aspects of waste management, AI technologies tackling what heath risks or health problems”. In this round of coding, these four coders review each other’s coding results to ensure consistency and accuracy. The senior coder reviewed the coding results of 94 articles, and discussed the definition and application scope of AI technologies with the four coders and formed consensus. In this round of coding, 13 articles were eliminated due to irrelevance of their contents. This review is based on the coding results of 81 articles.

The quality of reviewed studies is moderate to high. Among 81 reviewed studies, 55 (68%) were published in journals receiving a Citescore or Impact Factor and 26 (32.1%) were published in journals receiving a citescore over 10. Since the purpose of assessing bias is to estimate the quality of the studies rather than to correct the empirical findings of the reviewed studies. To save the research cost, we randomly select a subset of the reviewed studies for bias evaluation. One senior author read and evaluated 8 articles including both review and empirical studies against a checklist of evaluation criteria adapted for each study applying diverse research methods [30]. Six of these articles were rated as having high quality, all of which were published in journals receiving a cite score. Two articles were rated as having moderate quality. Six of the eight articles have moderate level of bias. We argue that the quality of reviewed studies is consistent with the quality of publication venues. (see Supplementary Appendix I).

## Results

### Application of AI technologies in stages of waste management

Tchobanoglous et al. [31] defined waste management as the effective supervision and handling, storage, collection, transportation, treatment and disposal of waste in a way that protects the environment and the public. AI technologies are applied to all stages of waste management (Figure 2).

**FIGURE 2:**
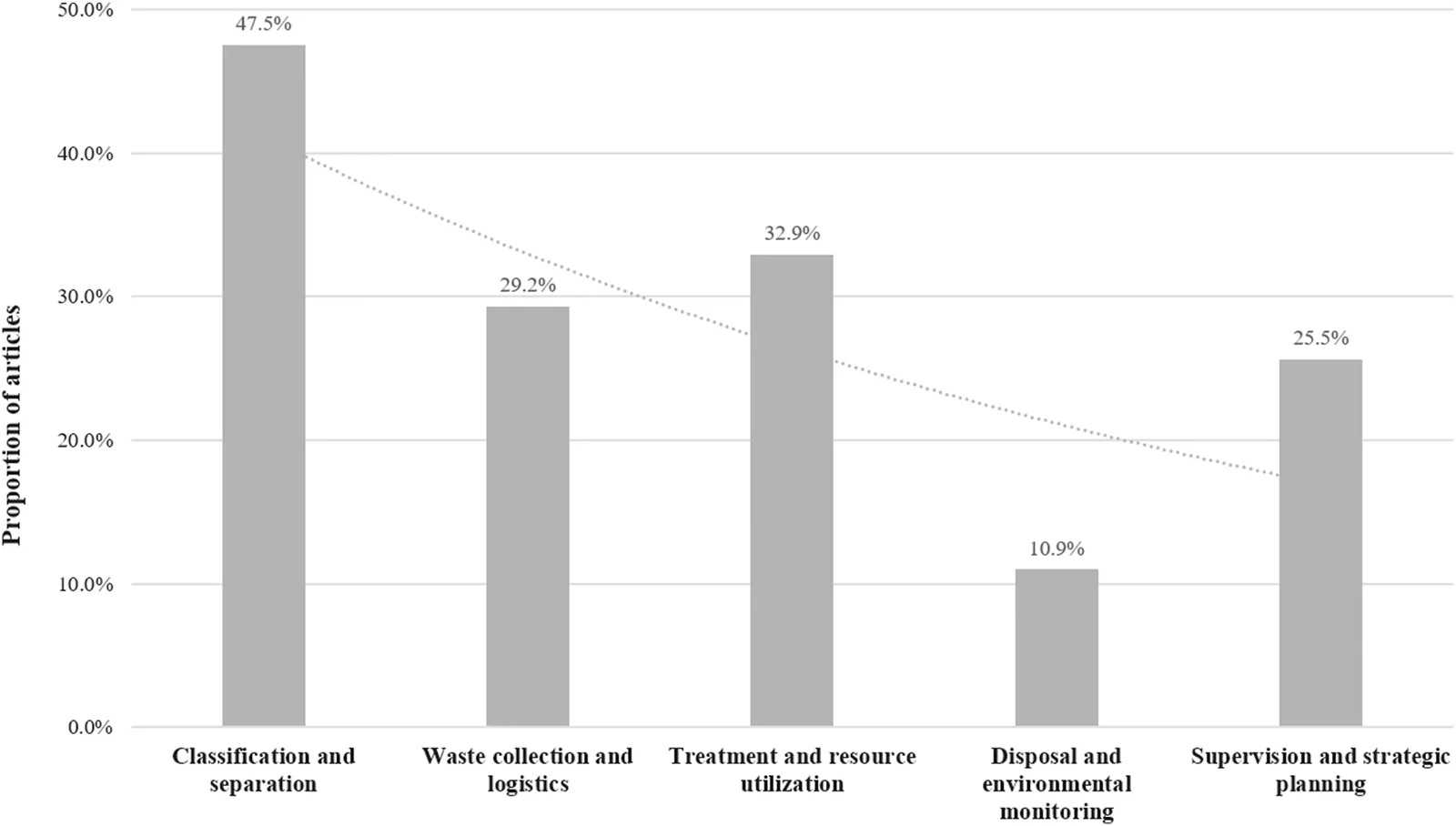
Distribution of reviewed articles by waste management stages (China, 2026).

The application of AI technologies is most extensive in the classification and separation stage of waste management, accounting for 47.5% (Table 1). This includes: 1) Utilizing computer vision, deep learning models, and sensor technologies to conduct real-time identification, classification, and automatic sorting of waste in recycling plants, on conveyor belts, or through smart trash bins; 2) Employing AI technologies at the source of waste generation (such as households, residential areas, and clinics) to monitor waste disposal and detecting misclassification.

**TABLE 1:** Waste management stage focus of reviewed articles (China, 2026).

Stages	Sub-stages	Proportion of articles
Classification and separation (47.5%)	Automatic and intelligent sorting	18.3%
	Source classification and management	29.2%
Waste collection and logistics (29.2%)	Collection route optimization	8.5%
	Logistics and facility network	8.5%
	Real-time status monitoring	12.2%
Treatment and resource utilization (32.9%)	Resource recycling technological innovation	14.6%
	Handling technical optimization	12.2%
	Waste-to-energy conversion	6.1%
Disposal and environmental monitoring (10.9%)	Final disposal management	3.7%
	Environmental compliance and impact monitoring	7.3%
Supervision and strategic planning (25.5%)	Waste generation and characteristic prediction	8.5%
	Strategic planning and system assessment	8.5%
	Risk management and social integration	8.5%

Some articles can be categorized in various stages and therefore, the percentages do not add up to 100%.

In the stage of waste collection and logistics, AI technologies have been applied to optimize the collection routes so as to balance efficiency and cost consideration [32, 33] (Table 1). Other studies focus on the optimization of the entire supply chain, such as the spatial optimization of recycling stations and the integration of the informal sector [34]. The Internet of Things (IoT) and remote sensing technologies have been utilized for compliance and safety monitoring (e.g., monitoring of waste bin overflow) [35, 36].

Research concerning the treatment and resource utilization stage focuses on converting waste into resources (Table 1), which is the key for developing a circular economy. Sabina et al.’s study utilizes AI and machine learning technologies to optimize the bioremediation and nutrient recovery strategies for agricultural and industrial waste [37]. Sathyabama et al. [38] uses Artificial Neural Network (ANN) technologies to predict the biogas production, optimizing the anaerobic digestion process. There are fewer studies concerning the conversion of waste into energy, but AI technologies have shown high potential to improve the efficiency of energy recovery [39, 40].

During the disposal and environmental monitoring stage, the research focus has shifted from process optimization to end-of-pipe control and risk prevention (Table 1). Yang et al. [41] addressed the groundwater pollution issue around landfill sites by using unsupervised machine learning methods to achieve pollution zoning and control. Fenton et al. [42] focused on the full-life-cycle management of chemicals, introducing AI for data analysis to enhance safety and minimize human and environmental exposure.

Finally, in the stage of supervision and strategic planning, AI technologies is utilized to support management decisions, focusing on system-level optimization and evaluation (Table 1). Some studies focus on the prediction of urban solid waste generation [43, 44]). Other studies, including reviews, cover the entire process of nuclear waste management, quantifying and warning of its potential environmental and health impacts [45, 46]. Al-Raeei [47] and Görçün et al. [48] explore the application of AI technologies in enhancing waste management strategies and industrial cleaning robots. Dinh, Fujiwara and Phu [34] study how to integrate the informal waste sector into the formal circular economy system.

### Types of AI technologies

AI means the study of “intelligent agents”: any device that observes its environment and takes actions to maximize the likelihood of achieving its goals. More broadly, a technology can be called AI when it can perform functions related to human thinking. Learning is an important aspect of AI [49]. Yevle and Mann [13] have analyzed in detail the development of AI technologies in waste management and classified them into supervised learning, unsupervised learning, and reinforcement learning techniques, and further divided them into different AI models, such as: machine learning, CNN (Convolutional Neural Network), transfer learning, hybrid/integrated learning, clustering methods, and deep reinforcement learning.

This article categorizes the AI technologies in the reviewed articles into four major categories and 14 specific technologies: supervised learning, unsupervised learning, reinforcement learning and deep learning. While like “machine learning” models, “deep learning” models employ both supervised and unsupervised learning techniques, it is identified as a separate coding category because of its ability to learn from mistakes and more efficiently achieve high level features from massive amount of data [50]. Coding “advantages of AI technologies” from reviewed articles reveals that AI technologies can address the deficiencies in previous waste management with new advantages (see Table 2).

**TABLE 2:** Artificial intelligence technologies and their advantages in waste management (China, 2026).

Categories	Specific techniques	Application/Advantages	Percentage of total reviewed articles
Supervised learning	• CNN and its variants • YOLO series • ResNet • Visual Geometry group (VGG) • MobileNet • Support vector machine (SVM) • Random forest (RF) • XGBoost • Transfer learning	• Realize real-time detection and high-precision classification of waste • Predict trends in municipal solid waste generation • Optimize treatment process parameters • Solve small sample problems and shorten model development cycles	39.5% (32/81)
Unsupervised learning	• K-means clustering • Principal component analysis (PCA) • Self-organizing map (SOM)	• Cluster data for accurate partitioning management • Identify potential patterns in waste composition • Use socio-economic data for waste characterization	4.9% (4/81)
Reinforcement learning	• Q-learning/Deep Q-network (DQN) • Adaptive evolution algorithms (e.g., RL-DE)	• Optimize catalyst selection and reaction conditions in the catalytic degradation of plastics • Adaptively tuning the hyperparameters of a waste classification model to improve the recognition accuracy and robustness of different waste streams	2.4% (2/81)
Deep learning	• Hybrid architecture (CNN + LSTM) • Transformer/self-attention modeling • Multi-model integration/fusion • Lightweight network design • Advanced computational frameworks (e.g., spherical fuzzy AHP)	• Simultaneously perform waste image classification (CNN) and gas production time series prediction (LSTM) • Significantly improve the segmentation and classification accuracy of disaster waste in complex scenarios through model integration • Designing lightweight models for high-speed and high-precision sorting at dumpster terminals • Processing subjective and objective criteria when evaluating smart waste management systems to assist in selecting the best solution	51.9% (42/81)

First, “supervised learning” refers to the process of learning the mapping between a set of input variables X and output variables Y, and applying this mapping to predict the output of unseen data [51]. In machine learning, it is also known as classification or inductive learning, similar to how humans learn from past experiences and acquire new knowledge to enhance our ability to solve current new problems [52]. In waste management, applying “supervised learning” technologies is to learn patterns from historical data, thereby accurately identifying or predicting new data. Articles involving supervised learning technologies total 32, accounting for 39%.

Second, unsupervised learning techniques represent specific input patterns in a way that reflects the statistical structure of the entire set of input patterns [53]. They discover the inherent structure or patterns from unlabeled data without the need for predefined categories, and are often used for data exploration, dimensionality reduction, and cluster analysis to identify waste composition characteristics, pollution sources, or for market segmentation, providing data-driven insights for management decisions. There are a total of 4 articles involving unsupervised learning, accounting for 4.9%.

Third, reinforcement learning lies between supervised learning and unsupervised learning, dealing with learning in sequential decision-making problems with limited feedback [54]. It focuses on learning through observing the environment and interacting with it, thus being conducive to achieving goals in uncertain and potentially complex environments [55]. Reinforcement learning pays great attention to sequential data rather than random input. The choice in the previous step will affect the subsequent development. It involves the concept of delayed rewards: rewards can only be obtained after completing the entire task [56]. The agent will autonomously learn the optimal decision strategy through continuous interaction with the environment based on the reward or punishment signals received. There are a total of 2 articles on reinforcement learning coding, accounting for 2.5%.

Fourth, deep learning is a comprehensive architecture that encompasses AI technologies that have not been explicitly classified into the above three categories. It enables computer models composed of multiple processing layers to learn data representations with multiple levels of abstraction. The lower layers near the data input learn simple features, while higher layers derive more complex features from the lower-level features, making it suitable for analyzing and extracting useful knowledge from a large amount of data from various sources [57]. The articles involved in the coding contain a total of 42 pieces, accounting for 51.9%.

The application of AI technologies has covered the entire cycle of waste management. As shown in Table 3, supervised learning and deep learning technologies run through the entire life cycle of waste from “classification” to “planning”. Specifically, supervised learning technologies represented by CNN mainly focus on the waste classification and separation stage, achieving high-precision and real-time identification and sorting of multiple types of waste. Meanwhile, the hybrid architecture and model integration technologies in deep learning have enhanced the ability to handle complex scenarios (such as post-disaster ruins, mixed waste streams) in waste management. In the collection, transportation and logistics stage, optimization algorithms like XGBoost and lightweight deep learning designs have become key technologies. The former directly improves operational efficiency and reduces costs by optimizing collection routes, while the latter becomes feasible and efficient based on real-time status monitoring through the Internet of Things.

**TABLE 3:** Artificial intelligence technologies involved in different waste management stages (China, 2026).

Stage	Sub-stages	Supervised learning	Unsupervised learning	Reinforcement learning	Deep learning
Classification and separation	Automatic and intelligent sorting	●	​	​	●
	Source classification and management	●	●	​	●
Waste collection and logistics	Collection route optimization	●	​	​	●
	Logistics and facility network	​	​	​	●
	Real-time status monitoring	●	​	​	●
Treatment and resource utilization	Resource recycling technological innovation	●	​	●	●
	Handling technical optimization	●	​	●	●
	Waste-to-energy conversion	​	​	​	●
Disposal and environmental monitoring	Final disposal management	​	●	​	​
	Environmental compliance and impact monitoring	​	​	​	●
Supervision and strategic planning	Waste generation and characteristic prediction	●	●	​	●
	Strategic planning and system assessment	​	​	​	●
	Risk management and social integration	​	​	​	●

“●” indicates that this artificial intelligence technology is present in the coded literature for this sub-stage.

It is worth noting that although reinforcement learning techniques are currently limited to the treatment and resource utilization stages, they have demonstrated significant potential in optimizing specific processes such as catalytic degradation of plastics [58]. At the strategic planning level, the need for an integrated decision-making framework is highlighted, with advanced computational frameworks within the realm of deep learning serving as the core tools for system evaluation and path design. These frameworks can handle uncertainties across multiple dimensions including the environment, economy, and society, providing a basis for sustainable system planning and a circular economy [59].

### AI technologies and health risks

Over 40% articles focus on how AI technologies mitigate health risks related to occupational exposure. Health outcomes are inferred from empirical studies rather than directly assessed via medical methods. Based on the content of coding item “what health risks and problems are targeted”, we identified five categories of health risks related to waste management that AI technologies have potential to tackle (see Table 4):infectious risk (9.9%), chemical toxicity risk (27.2%), physical injury risk (3.7%), occupational exposure risk (42%), and general public health risk (21%) (Table 4).

**TABLE 4:** Artificial intelligence technologies for health risk management (China, 2026).

Types of health risks	Types of wastes/scenarios	Affected population	AI technologies	Waste management stages
Infectious risk	• Infectious municipal waste • Biomedical waste	Waste workers, healthcare workers, general public	• Supervised learning (CNN and its variants) • Deep learning (hybrid architecture)	• Classification and separation • Treatment and resource utilization
Chemical toxicity risk	• E-waste • Industrial chemicals • Hazardous waste	Demolition workers, informal recyclers, residents of surrounding communities	Supervised learning (SVM, XGBoost, CNN, etc.)	• Classification and separation • Disposal and environmental monitoring
Physical injury risk	• Construction demolition waste • Household waste • Mechanical operating environment	Sorting workers, collection workers	Deep learning	• Classification and separation • Waste collection and logistics
Long-term occupational exposure risk	• Landfills • Mixed waste sorting lines • Informal recycling stations	Landfill workers, sorters, informal sector workers	• Unsupervised learning (K-means, SOM) • Supervised learning • Deep learning	• Classification and separation • Disposal and environmental monitoring

AI technologies can be deployed to deliver health benefits to waste management practitioners and the general public. The applications of AI for this purpose throughout the life cycle of waste management are reflected in the following dimensions.

First, AI enables the automated identification and precise isolation of risk sources. Equipped with image recognition technology, AI systems can automatically and accurately identify wastes posing specific health hazards, including infectious medical waste [8], electronic components containing hazardous chemicals, and damaged chemical containers [41]. This allows the system to automatically sort and isolate hazardous waste prior to manual processing, eliminating direct human contact and exposure risks inherent in traditional manual sorting workflows.

Second, AI-powered robots replace human labor in high-risk operational scenarios. Repetitive, high-exposure tasks such as waste sorting, crushing, and transportation can be autonomously performed by AI-driven robotic arms and automated production lines [60]. This effectively mitigates a wide range of occupational hazards, including physical cuts, biological infections, and dust inhalation. More importantly, it transforms the operational interaction model: workers are relocated to back-end system monitoring and management roles, significantly improving the overall safety of waste management operations [61].

Third, supervised learning and deep learning technologies support real-time environmental risk identification and dynamic early warning mechanisms. Based on sensor networks deployed at landfills, processing workshops and other work sites [44], AI systems can continuously monitor real-time indicators including air quality, toxic gas concentration, and leachate leakage. Combined with predictive analytical models, the systems can issue timely early warnings of abnormal conditions. This empowers managers to adjust operational plans, evacuate on-site personnel and launch emergency responses in a timely manner, proactively preventing workers from exposure to sudden, high-concentration toxic hazards.

Fourth, unsupervised learning enables systematic occupational exposure analysis and targeted intervention. AI can mine long-term datasets including environmental monitoring records, worker health risk profiles, and operational logs to identify latent risk patterns and high-risk zones associated with chronic occupational diseases. For instance, pollution dispersion simulation and cluster analysis can assist in delineating management zones with differentiated risk levels [62]. This provides a scientific basis for formulating tiered protection standards, optimizing facility layout, and arranging working hours rationally. From a systematic planning perspective, such interventions effectively reduce long-term health risks for all frontline occupational groups.

## Discussion

Based on the findings, this section discusses the policy, regulatory, and governance innovations required for responsible AI deployment, followed by implications for the waste management industry.

The transformative potential of AI in waste management-including efficiency gains, risk reduction, and public health protection-cannot be fully realized without supportive policies, adaptive regulations, and socio-technical governance. Effective policies, regulations and governance approaches operate across multiple scales: national strategic planning, municipal implementation rules, and cross-regional or international coordination [46, 63]. Four interrelated policy and regulatory priorities emerge from reviewed literature to guide AI for good in waste management.

First, establishing regulations and standards to support the application of AI technologies. In the context of Industry 4.0, large-scale data collection requires a corresponding data privacy framework [39] to ensure that the information collected by smart trash cans, sensor networks and other devices is not misused. At the same time, governments should take the lead in formulating unified technical standards for garbage classification and treatment, providing a standardized data environment for the training and deployment of artificial intelligence algorithms. For example, establishing a strict national garbage classification policy [64] to change the current loose enforcement of laws and regulations.

Second, innovating policies and regulations for high-risk waste streams. Specialized regulation is essential for hazardous waste categories. For e-waste, extended producer responsibility (EPR) should be legally mandated [7], holding manufacturers accountable for end-of-life recycling while leveraging AI to improve sorting and dismantling efficiency [65, 66]. The management of plastic waste requires strengthening the control of disposable plastics, encouraging the research and development of degradable materials [67], and using machine learning to optimize the catalytic degradation process [68].

Third, designing policy tools that emphasize both incentives and supervision. On the one hand, the government can encourage enterprises and communities to adopt AI-driven garbage treatment technologies through tax incentives, financial subsidies, and green procurement [64, 69]. On the other hand, it is necessary to establish a punishment and accountability mechanism, such as cross-regional law enforcement against illegal dumping [63], and improving regulatory capabilities by using remote sensing and image recognition technologies.

Fourth, building an inclusive and equitable AI-empowered waste management system. Some literature emphasizes the necessity of integrating the informal recycling sector into the formal system [34], advocating for a just transition through spatial optimization, occupational health training, and social insurance. Introduction of new AI and waste infrastructure projects requires community and informal workers’ input to forecast livelihood risks and design retraining programs. Governments need to fund local-language digital literacy and AI operation training for informal waste workers, enabling them to transition into AI sorting oversight, waste inspection and emission monitoring roles instead of being eliminated by automation [70, 71].

Furthermore, public health literature largely ignores the public health benefits generated by technological innovations within waste management systems. This article’s interdisciplinary lens helps policymakers expand their public health intervention strategies and attach greater importance to deploying AI in waste management as a public health instrument.

To accelerate the safe, healthy, and sustainable development of the waste management sector, we propose the following industry-level recommendations.

First, accelerating the deployment of health-oriented waste management systems. The industry should gradually shift from efficiency-dominated design to health-oriented system development, prioritizing AI applications that reduce occupational exposure, improve workplace safety, and protect community health. Demonstration projects for AI-integrated sorting, robotic handling, and real-time risk monitoring should be scaled up in high-risk scenarios such as landfill sites, waste-sorting facilities, and informal recycling zones.

Second, strengthening cross-sector collaboration between technology research institutions and companies, waste management enterprises, and public health authorities. Joint and interdisciplinary research and testing platforms should be established to improve the robustness, adaptability, and practicality of AI models under real-world waste scenarios, particularly in developing regions and disaster-affected areas.

Third, promoting the digital upgrading of the waste management industrial chain. This includes the smart renovation of waste collection networks, monitoring of transportation and storage, waste treatment and resource utilization processes, and full-life-cycle risk management for waste disposal.

Fourth, enhancing occupational health protection and skill upgrading for workers. As automation increases, training programs should be provided to help frontline staff transit to roles of equipment operation, system supervision, and maintenance. Health monitoring, risk awareness, and standardized protection protocols should be integrated into industrial daily operation.

Fifth, advancing circular economy goals through AI-driven data governance. According to UNEP report [72], a Circular Economy approach will generate projected annual full net gains worthy of USD 108.5billion. AI can be deployed to support accurate waste characterization, demand forecasting, and material flow tracking, improving the stability and quality of recycled resources. Extended Producer Responsibility systems can be enhanced with AI-enabled monitoring and evaluation to improve accountability and recycling performance.

### Conclusion

This systematic review provides a preliminary overview of the research landscape of AI application for mitigating health risks in the cycle of waste management, as well as the indispensable role of institutional innovation.

Rapid global growth in solid waste poses severe threats to ecological security and public health, while conventional waste management suffers from low sorting efficiency, high occupational exposure, weak adaptability to complex scenarios, and insufficient health protection [1, 57]. Driven by the paradigm shift from end-of-pipe disposal to whole-life-cycle collaborative governance, AI has evolved from expert systems and predictive models to deep learning models that enabled intelligent sorting, route optimization, resource recovery, and environmental risk early warning. Future research should further investigate how unsupervised and reinforcement learning can improve waste management performance from the perspective of mitigating occupational and public health risks.

As demonstrated in this review, AI applications cover classification, collection, treatment, disposal, and strategic planning, significantly improving accuracy, efficiency, and resource utilization while reducing direct physical contact, infectious risks, chemical toxicity, and occupational exposure for workers and communities [11, 12]. In the future, more research is needed on how AI technologies may address the infectious risks and physical injury risks associated with waste management operations, Studies on the application of AI technologies at the stage of disposal and environmental monitoring need to be strengthened.

A key contribution of this study is to highlight that AI for good in waste management is not merely a technical achievement but a socio-technical transformation that integrates public values of efficiency, health and equity. AI delivers these values by automating high-risk operations, enabling precise risk source identification, supporting real-time environmental monitoring, and facilitating occupational health interventions. Such progress aligns with the ethical mission of responsible innovation: technology should protect vulnerable groups, reduce environmental injustice, and serve public health rather than only pursue economic returns [13].

Equally importantly, policy, regulation, and governance innovations are prerequisites for translating AI potential into AI for social good while preventing the emerging risks of hazards stemming from AI’s application to data analysis and decision-making [73]. Without supportive institutions, even advanced algorithms cannot achieve scalable, safe, and equitable deployment. This review identifies three institutional imperatives:

First, technical standards and data privacy regimes must be established to ensure reliable, transparent, and secure AI applications.

Second, targeted policies for high-risk waste streams enhance health protection and circularity.

Third, incentive-and-oversight policy mixes, together with inclusive institutions for informal waste workers, ensure a just transition and prevent new forms of inequity.

In summary, targeted AI interventions have great potential to reshape waste management workflows away from labor-intensive, high-exposure operations and introduce health-protective capabilities. Scholars can engage with this research agenda by adopting medical research methodologies to quantify the health risk reductions delivered by technological innovations in waste management. To further realize AI for social and environmental good, continuous technological innovation must be matched with policy, regulatory and collaborative governance innovation that encourages financial investment and strengthen public trust, with sustained attention to the needs of frontline workers and marginalized communities.

The study has the following limitations. It does not systematically distinguish the mature AI technologies from conceptual prototype technologies. It also includes many review and conceptual studies that do not report measurable outcome of AI technologies. The quality of reviewed studies varies in respect of transparent reporting, cross-validation of data analysis, discussion of contradictory findings, model robustness testing and so on (see Supplementary Appendix I). The environmental and health risks posed by AI devices—including growing e-waste, high energy consumption and noise pollution—have also been understudied in the reviewed literature. Grey literature such as pre-prints, policy documents, guidelines and research reports by think tanks and consultants are not included in the review. Future review studies can be focused on resource, and institutional barrier of applying AI for health-oriented waste management such as infrastructure limitations, data availability, cybersecurity risks, regulatory fragmentation, and deployment cost in low-resource settings.
