# Supplementary material for: Deploying artificial intelligence for occupational and environmental health in waste management: a systematic review
Source: Int J Public Health. 2026 Sep 21;71:1610020. doi: 10.3389/ijph.2026.1610020 (PMC13634977; doi:10.3389/ijph.2026.1610020)
Supplement: Supplementary file 1 [file Table1.docx]

Appendix I

**Table 1 Quality of Reviewed Studies and Risk of Bias**

|  | Article Title | Publication Venue | Research Design | Quality Assessment | Risk of Bias |
| --- | --- | --- | --- | --- | --- |
| 1 | Application of the vision-based deep learning technique for waste classification using the robotic manipulation system | International Journal of Cognitive Computing in Engineering  (CiteScore 18, year 2025) | Applied engineering experimental study | High: Clear research objectives & systematic workflow; Standardized technical methods;Controlled repeated experiments;self-collected validated dataset; transparent reporting. Full industrial applicability is low. | Moderate: limited experimental scenarios, small dataset and lack of robustness testing. |
| 2 | Treatment options of nitrogen heterocyclic compounds in industrial wastewater: From fundamental technologies to energy valorization applications and future process design strategies | Water Research Journal (CiteScore 21.6, year 2025, SCIE) | Review study | High: Comprehensive & Systematic Literature Review, In-depth Mechanism & Comparative Analysis; High Practical & Industrial Value; In-depth discussion on AI. | Moderate: unclear about the databases and  inclusion criteria for literature; Selective reporting of technical indicators;Inconsistent data standardization;Weak discussion on conflicting research findings |
| 3 | Recycling of E-waste and Green Electronic Manufacturing | E3S Web of Conference | Review study | Moderate: Transparent Research Methodology; Multi-dimensional Results Presentation; Contradictory annual growth rate;Descriptive dominance; Lack of in-depth interpretation;Undefined database. | Moderate: literature selection bias (the unknown database and potential language restrictions); the existence of data statistical error. |
| 4 | Machine Learning Techniques for Predicting the Increase in Municipal Solid Waste Generation in Lima, Perú | 4th IEEE International Conference on AI in Cybersecurity | Applied quantitative empirical study combining data-driven machine learning predictive modelling, with a retrospective observational design | Moderate: Clear research objectives & practical value;Reasonable model selection and standardized evaluation;Multi-source official data adoption; Small and constrained dataset; Simplified missing data handling;Absence of data balancing and cross-validation;Limited discussion of external validity | Moderate: Data and model overfitting risk; Unverified model robustness; the study does not conduct multicollinearity testing or control for confounding effects;the model trained on discontinuous time data may produce biased predictions for continuous future waste trends. |
| 5 | Exploring the potential of artificial intelligence in nuclear waste management: Applications, challenges, and future directions | Nuclear Engineering and Design Journal (CiteScore 3.6, year 2025,SCIE) | Review | High: Comprehensive Literature Integration & Citation；Balanced Discussion of Advantages and Challenges;Linking technical analysis to industrial demands; Lack of Quantitative Analysis | Low: publication bias favoring positive results and successful AI application cases |
| 6 | Artificial intelligence for climate resilience: advancing sustainable goals in SDGs 11 and 13 and its relationship to pandemics | Discover Sustainability Journal (CiteScore 2.6, year 2024，ESCI） | Review | High: Clear, focused research scope and structured framework;Transparent, comprehensive literature search protocol;Rigorous qualitative thematic analysis design;Balanced critical analysis | Moderate: no systematic review protocol; Single-author screening without duplicate independent review |
| 7 | Precise management and control around the landfill integrating artificial intelligence and groundwater pollution risks | Chemosphere Journal (CiteScore 21.1, year 2025, SCIE) | Mixed-method quantitative empirical case study design | High: multi-method framework; transparent field sampling & laboratory analytical protocols; advanced uncertainty & sensitivity analysis;explicitly compares model strengths | Low: including neutral/unfavorable results;Single-site case creates contextual bias |
| 8 | Artificial Intelligence in enhancing sustainable practices for infectious municipal waste classification | Waste Management Journal (CiteScore14,year 2025, SCIE) | Applied algorithmic experimental research | High: Clear, Novel Research Gap Identification;Two independent datasets are used for cross-validation;Transparent, Reproducible Methodological Framework;Multi-Dimensional Performance Validation | Moderate: Geographic sampling restriction;training data distribution deviates from real operational data; performance-comparison risk. |
